# Scarring and Skin Fibrosis Reversal with Regenerative Surgery and Stem Cell Therapy

**DOI:** 10.3390/cells13050443

**Published:** 2024-03-03

**Authors:** Aurora Almadori, Peter EM Butler

**Affiliations:** 1 Centre for Nanotechnology and Regenerative Medicine, Division of Surgery & Interventional Science, University College of London, London NW3 2QG, UK; peter.butler@ucl.ac.uk; 2 Department of Plastic Surgery, Royal Free London NHS Foundation Trust Hospital, London NW3 2QG, UK; 3 The Charles Wolfson Centre for Reconstructive Surgery, Royal Free Hospital Campus, University College of London, London NW3 2QG, UK

**Keywords:** cell therapy, regenerative medicine, regenerative surgery, adipose stem cells, stromal vascular fraction, extracellular matrix, fat grafting, lipofilling, lipotransfer, fibrosis, scar

## Abstract

Skin scarring and fibrosis affect millions of people worldwide, representing a serious clinical problem causing physical and psychological challenges for patients. Stem cell therapy and regenerative surgery represent a new area of treatment focused on promoting the body’s natural ability to repair damaged tissue. Adipose-derived stem cells (ASCs) represent an optimal choice for practical regenerative medicine due to their abundance, autologous tissue origin, non-immunogenicity, and ease of access with minimal morbidity for patients. This review of the literature explores the current body of evidence around the use of ASCs-based regenerative strategies for the treatment of scarring and skin fibrosis, exploring the different surgical approaches and their application in multiple fibrotic skin conditions. Human, animal, and in vitro studies demonstrate that ASCs present potentialities in modifying scar tissue and fibrosis by suppressing extracellular matrix (ECM) synthesis and promoting the degradation of their constituents. Through softening skin fibrosis, function and overall quality of life may be considerably enhanced in different patient cohorts presenting with scar-related symptoms. The use of stem cell therapies for skin scar repair and regeneration represents a paradigm shift, offering potential alternative therapeutic avenues for fibrosis, a condition that currently lacks a cure.

## 1. Introduction

### 1.1. Cellular Dysfunction in Fibrosis and Scarring

Fibrosis represents a major burden affecting millions of people worldwide [1], with significant morbidity and mortality. It is estimated that 45% of deaths in the western world can be attributed to diseases where fibrosis plays a major etiological role [2].

Scarring and fibrosis are hallmarks of a heterogeneous group of disorders that can develop in all tissues or organs [3]. It manifests as a spectrum of fibrous connective tissue deposition from limited areas, such as excessive accumulation of in and around inflamed or damaged tissue, leading to permanent scarring, organ malfunction, and, potentially, death. Dermal fibrosis is excessive scarring of the skin, and it is a consequence of an exaggerated healing response, particularly disproportionate fibroblast proliferation and extracellular matrix (ECM) production in the dermis [4]. Although scar tissue formation is a critical aspect of the wound healing process and tissue repair, triggered by various factors such as infection, inflammation, autoimmune disorders, degenerative diseases, tumors, and injuries, the development of a pathological, chronic wound healing response presents significant challenges for patients, both physically and psychologically [5,6].

**Figure 1:**
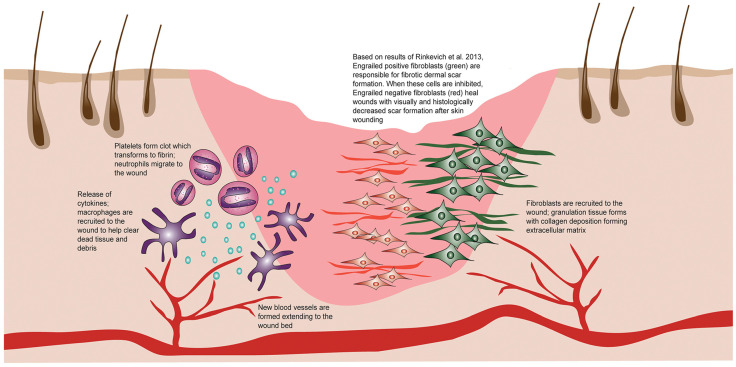
The wound healing response. The stages of wound healing following injury involve hemostasis, inflammation, proliferation, and remodeling. Fibroblasts play a crucial role in the formation of cutaneous scars post-injury. Inhibition of these cells leads to a more regenerative phenotype, resulting in reduced scarring. Reproduced with permission from Jones et al., Transfusions, published by Wiley, 2019 [7].

The development of skin fibrosis can result from an abnormal reaction to a single significant injury, although more frequently, it occurs in response to persistent or repetitive injuries [7]. Regardless of the site of injury, type of tissue, or nature of the injury, the healing process typically involves three main phases: inflammation, proliferation, and remodeling (Figure 1).

**Figure 2:**
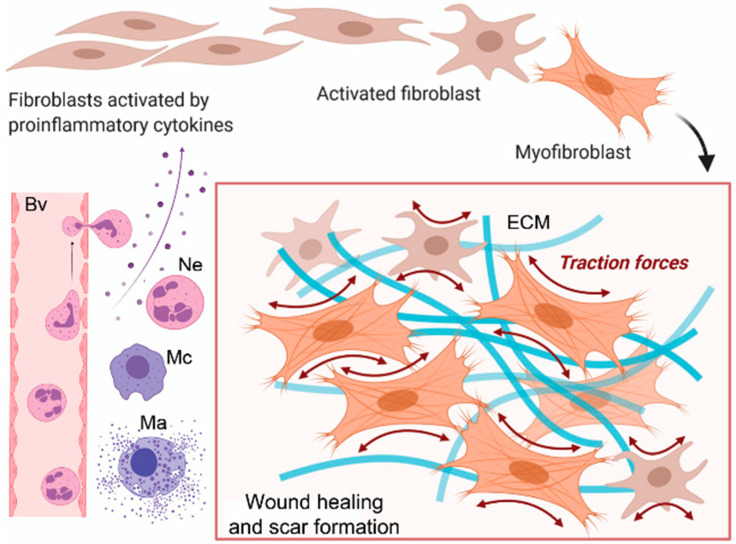
Cellular mechanism of skin fibrosis. Fibroblast activation plays a critical role in the development of fibrosis. A blood vessel (Bv), neutrophils (Ne), macrophages (Mc), and mast cells (Ma) are indicated. ECM: extracellular matrix. Reproduced from Fertala et al., Biomolecules; published by MDPI, 2023 [11].

Following acute or systemic injury, inflammatory cells derived from blood and local sources migrate to the damaged sites, initiating the tissue repair process by secreting numerous growth factors. These growth factors, in conjunction with mechanical stress and adhesion proteins, then activate both resident and migratory fibroblastic cells [8]. The activated form of fibroblast, the myofibroblasts, increase the secretion of chemical signals, such as cytokines, chemokines, and growth factors, with a double effect: on the one hand, they stimulate the excessive accumulation of ECM components, including fibrillar collagens, fibronectin, proteoglycans, glycosaminoglycans, and hyaluronic acid, at the site of tissue injury, while on the other hand, they suppress the activity of the matrix-metalloproteinases (MMPs), a family of zinc-dependent metalloendopeptidases that can degrade or cleave many components of the ECM, as well as a wide range of other extracellular proteins [9,10]. This combined effect causes abnormal ECM deposition, resulting in increased tissue stiffness [9]. While prompt and efficient healing of skin wounds is essential to maintaining the vital barrier function of the skin, a pathological wound healing response can result in abnormal extracellular matrix (ECM) deposition and diminished degradation, ultimately leading to scarring and fibrosis (Figure 2).

Skin scarring, the most common outcome of cutaneous fibrosis, can emerge after traumatic injury, iatrogenic conditions, or autoimmune diseases. It represents a significant clinical challenge, with physical and psychological implications for patients [6,12]. Globally, millions of people present with aberrant skin wound repair, culminating in pathological scarring and fibrosis; each year, eleven million people are affected solely by burn injuries [13,14,15], with 71% of these individuals experiencing significant scarring and fibrosis [16]. Despite these alarming statistics, effective cures or therapies for adverse scarring are still lacking.

### 1.2. Conventional Therapies for Fibrosis and Scarring

Despite substantial efforts by the scientific community to find a cure for fibrosis (about 3000 clinical trials on fibrosis and scarring are currently listed on clinicaltrials.gov), an effective cure is still lacking [17].

Multiple approaches have been proposed to treat dermal fibrosis and scarring after burn injuries, with different levels of results. Often, patients need a combination of different options because these therapies might not be effective on their own [18]. Non-invasive options include the use of compressive therapy, scar massage with oils or creams, and silicone sheets. However, evidence on these options is limited [18]. A meta-analysis showed that compression therapy induces only a minor reduction in scar height [19], and the optimal pressure has to be defined; evidence for scar massage is also weak, with a recent meta-analysis showing only anecdotal evidence for effectiveness [20]; results on the use of silicone applied topically appear to be contradictory regarding post-hypertrophic scar prevention [21].

More invasive approaches encompass surgical revision, involving the excision of the scar tissue followed by margin re-suturing, intralesional corticosteroid injections, and laser therapy. Intralesional corticosteroids modulate collagen synthesis, fibroblasts, and keratinocyte proliferation and suppress *TGF-β*. However, its use is associated with complications such as dermal thinning, fat atrophy, and pigment changes [22]. Pulsed dye laser, ablative fractional CO_2_ laser, and intense pulsed light have been suggested to target specific aspects of hypertrophic scars, such as collagen remodeling, improved vascularity, reduced height, increased pliability, and addressing colors and abnormal textures [22]. However, while laser therapy presents a promising avenue for enhancing scar quality post-burns, further foundational research and clinical trials are necessary. Additional treatment modalities with varying degrees of success include fluorouracil, interferon gamma, bleomycin injections, radiotherapy, cryosurgery, and micro-needling.

Among these, micro-needling showed encouraging results in scars. The rationale is that multiple micro-perforations of the stratum corneum produce micro-wounds in the epidermis and papillary dermis, stimulating collagen production. Nevertheless, none of these treatment modalities have shown consistently satisfactory efficacy, leaving patients with scars still struggling with significant concerns related to appearance and functional contractures. Hence, finding a definitive cure for scarring and fibrosis remains a significant challenge [22].

### 1.3. Cellular Therapy for Fibrosis and Scarring

Among the cellular therapies currently available, the use of stem cells has become increasingly popular. An increasing body of evidence on stem cell therapies showcases remarkable potential as groundbreaking treatments for a range of conditions, among them fibrosis and scarring. [23,24]. Stem cell therapy and regenerative surgery represent a new area of treatment focused on promoting the body’s natural ability to repair damaged tissue. The potential use of stem cell therapies for the repair and regeneration of various tissues and organs offers a paradigm shift that may provide alternative therapeutic solutions for several diseases [25].

The clinical use of either embryonic stem cells or induced pluripotent stem cells remains limited because of cell regulations, ethical considerations, and the requirement for genetic manipulation [25]. Adult autologous mesenchymal stem cells (MSCs) do not present these ethical issues and have been successfully explored in clinical studies on fibrosis, particularly to treat liver cirrhosis [26], idiopathic pulmonary fibrosis [27], myocardial fibrosis [28], renal fibrosis [29], and to repair pulmonary tissue that had been injured by thoracic irradiation [30]. Among the different sources of MSCs, bone marrow has been the most commonly adopted, containing a population of MSCs called bone marrow stem cells (BMSCs). However, their use presents drawbacks: they are extracted by a painful process that may cause donor site morbidity in the patient, and because the retrieved marrow has less of them, ex vivo expansion is frequently required [31]. Adipose tissue represents an attractive alternative source of MSCs as it is easily collected via a liposuction operation in large volumes and is abundant with MSCs called adipose-derived stem cells (ASCs) [31,32,33,34]. Moreover, the donor site treatment, which involves removing excess adipose tissue, is well tolerated and often welcomed by the patient. The ASCs therefore appear to be an ideal population of stem cells for practical regenerative medicine, given that they are plentiful, of autologous tissue origin and thus non-immunogenic, and are more easily available with minimal morbidity for patients [25]. In addition to that, the ASCs seem to be more efficient in reducing skin fibrosis than the BMSCs, with evidence showing that the ASCs were significantly more efficient than BMSCs in reducing *TNFα* and *IL1β* and enhancing the ratio of *MMP1/TIMP1* [35].

## 2. Aim of the Study

With this narrative review of the literature, we aimed at underpinning and summarizing the current body of evidence around the use of adipose stem cells-based regenerative strategies for the treatment of scarring and skin fibrosis, exploring the different surgical approaches used and the fibrotic conditions successfully treated.

## 3. Types of Adipose Stem Cell-Based Therapies

### 3.1. Fat Grafting

Autologous fat grafting (FG) consists of removing adipose tissue from areas of the body where it is in excess, processing it, and injecting the processed lipoaspirate into the area to be treated. This procedure gained popularity following the recognition of adipose tissue as a source of adipose-derived stem cells (ASCs) and its subsequent integration into standard clinical practice for reconstructing soft tissue defects resulting from cancer resection, trauma, and chronic wounds. Fat grafting (FG) primarily consists of mature adipocytes, pre-adipocytes, stem cells, and growth factors. Due to its biocompatibility, minimal invasiveness, widespread availability, lack of immunogenicity, and substantial regenerative potential, FG is now regarded as the ideal soft tissue filler [36]. Its use has been proposed in numerous clinical applications aimed at volumetric augmentation and tissue regeneration, including the treatment of scars and fibrosis.

**Figure 3:**
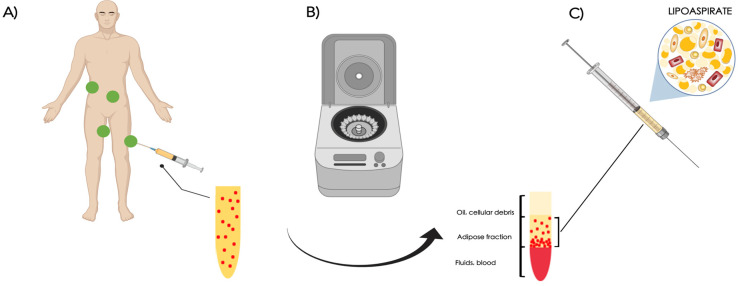
Fat grafting technique. The adipose tissue is harvested from the abdomen, inner thighs, knees, or hips (**A**); it is processed via centrifugation at 3000 rpm per 3 minutes to concentrate the fraction rich in ASCs (**B**); after discarding the upper and lower parts, the purified adipose tissue rich with ASCs is then available to be grafted in the recipient site (**C**).

Multiple surgical methods have been proposed in order to maximize the final product. The quantity, viability, and differentiation potential of the ASCs, as well as the lipoaspirate’s viscosity, can all be impacted by the different methods used for harvesting, processing, and infiltration. The method described by Coleman is considered the standard fat grafting (Figure 3).

The rationale of this treatment in scarred and fibrotic tissues consists in (1) direct replacement of the subcutaneous volume loss in the depressed scar where a loss of subcutaneous fat is appreciable, (2) physical release of the tethered injured skin/scar from the underlying tissues (‘padding effect’), and (3) improvement in scar quality via tissue remodeling and regeneration. The latter is due to the regenerative properties of the ASCs. There are three stages to the procedure: the first involves removing fat from a donor location (Figure 3A). Hips, inner thighs, the abdomen, and the knees are typically good donor sites. The extracted fat is processed in the second phase, which involves centrifuging it at 3000 rpm for three minutes (Figure 3B). Centrifugation is used to concentrate the fraction rich in ASCs while also eliminating unwanted components like blood, oil, and cellular debris. This procedure purifies the fat, making it ready for the third and last step—grafting it onto the face, hand, trunk, limbs, or anywhere else it is needed (Figure 3C). Centrifugation can be substituted with washing, filtering, or decanting the fat.

### 3.2. ASCs and SVF

**Figure 4:**
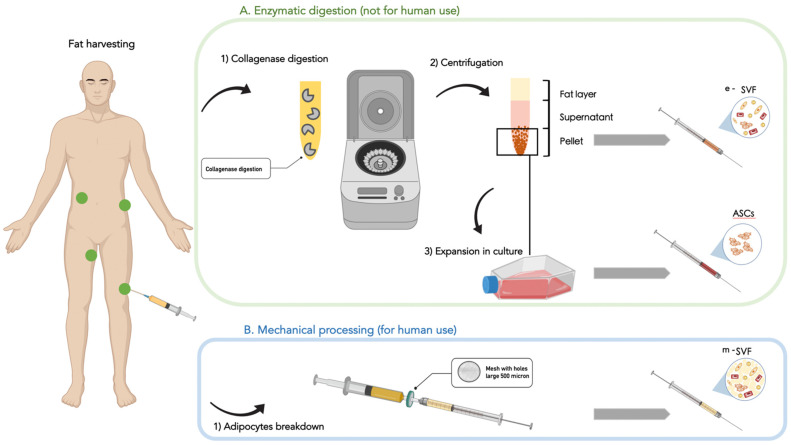
SVF and ASCs. The diagram illustrates different options for processing the adipose tissue to obtain progenitors cells (SVF, stromal vascular fraction, or ASCs, adipose-derived stem cells). Option (**A**) (in green) shows the passages that start with collagenase digestion (1) leading to enzymatically obtained SVF (e-SVF), which is a heterogeneous cell population mainly composed of ASCs, perivascular cells, endothelial cells, inflammatory cells, cell debris, and erythrocytes obtained from the lipoaspirate after collagenase digestion. After culture expansion (3), e-SVF yields a homogeneous population of plastic-adherent cells, the ASCs, that are described as CD31−, CD34+, CD45−, CD90+, CD105−, and CD146−. Option (**B**) (in blue) shows an alternative method to select mechanically obtained SVF (m-SVF) suspended in a solution mainly composed of broken adipocytes and cell debris.

Regenerative products derived from adipose tissue can be readily obtained through lipoaspiration and offer a substantial yield of multipotent cells (Figure 4).

These include stromal vascular fraction (SVF) and adipose-derived stem cells (ASCs). SVF is a heterogeneous cell population mainly composed of ASCs, perivascular cells, endothelial cells, inflammatory cells, cell debris, and erythrocytes. It can be obtained from the lipoaspirate after collagenase digestion (Figure 4A). After culture expansion (Figure 4A), SVF yields a homogeneous population of plastic-adherent cells, the ASCs, that are described as CD31−, CD34+, CD45−, CD90+, CD105−, and CD146− (Figure 4A).

Several methods have been outlined for extracting the stem cell component and administering it either independently or in conjunction with the lipoaspirate at the target site. However, these methods necessitate specialized equipment for adipose tissue storage, treatment with chemical agents like collagenase, and in vitro expansion of adipose-derived stem cells (ASCs) before re-implantation. Such procedures are prohibited by the majority of regulatory frameworks, are costly, and are frequently impractical for routine clinical use. For these reasons, mechanical methods to concentrate the SVF, such as fat emulsification (also called nanofat), are currently being explored because they do not require laboratory facilities and comply with current regulations on tissue manipulation for human use (Figure 4B). Emulsified fat is a technique introduced by Tonnard and represents an alternative to more complicated SVF isolation protocols [37].

The quantity of SVF cells obtained from nanofat is comparable to enzymatic methods, while requiring less fat tissue intake and without involving any substantial tissue manipulation [38].

### 3.3. Combined Approaches

#### Fat Grafting Mixed with PRP

Efforts to ‘enrich’ fat grafting involved the addition of platelet-rich plasma (PRP). This strategy aims to enhance the vascularization and viability of the graft by leveraging the growth factors and blood products present in PRP, which interact with surrounding cells and promote adipogenesis. However, clinical results in terms of increased volumetric survival of fat grafting when mixed with PRP are contradictory, as in some studies, PRP increased the fat survival rate while in others, it did not [39]. PRP, known since the 1970s, has been utilized clinically across various pathologies due to its ability to stimulate tissue repair and regeneration processes. These therapeutic properties stem from its high concentrations of growth factors, including *PDGF*, *TGF-β*, *IGF*, *EGF*, *FGF-2*, and *CTGF*. PRP has been demonstrated to enhance cell proliferation, collagen production, angiogenesis, and *MMPs* 1 and 3, leading to cellular and hormonal activation akin to the inflammatory phase of the healing process.

**Figure 5:**
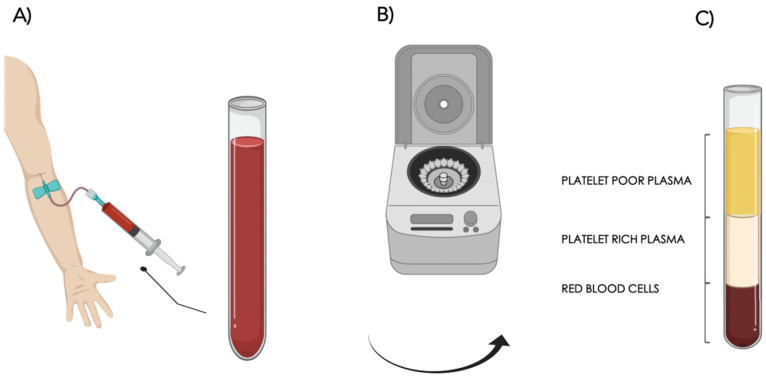
PRP consists of peripheral blood collection (**A**); centrifugation to separate the blood into different components (**B**); and selection of the fraction of plasma rich with platelets for injection at the recipient site (**C**).

PRP administration involves the collection of blood, typically through a syringe and needle in the arm (Figure 5A). The volume of blood required depends on the size of the treatment area and the desired platelet concentration. Following collection, the blood is centrifuged (Figure 5B) to separate it into three components: plasma (the watery portion), the PRP layer, and the cellular layer containing red and white blood cells (Figure 5C). The PRP layer is then extracted for clinical use. Subsequently, after local anesthesia is applied, the PRP is injected into the injured tissue. The primary indication for PRP therapy is to achieve a regenerative effect through a minimally invasive outpatient procedure. While PRP does not provide a volumetric filling effect, its purpose is to enhance scarring when used in conjunction with fat grafting.

## 4. Application in Fibrotic Skin Conditions

**Figure 6:**
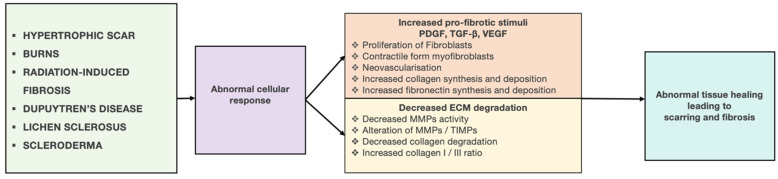
Commonalities among different fibrotic skin conditions successfully treated with ASCs-based therapies.

Adipose stem cells-based therapies have been successfully used to reverse dermal fibrosis and scarring in multiple fibrotic skin conditions (Figure 6).

### 4.1. Hypertrophic Scars

Hypertrophic scars are common complications of different tissue injuries. Excessive ECM combined with inadequate remodeling of scar tissue results in an aesthetically and functionally unsatisfactory, painful, pruritic scar that can impair function. A recent meta-analysis, including nine RCTs on fat grafting for hypertrophic scars, showed that this treatment is successful in reducing scars [40]. In comparison to a saline injection control group, patients undergoing fat grafting exhibited a higher rate of scar healing [OR = 3.54, 95% CI (1.50–8.39), *p* = 0.004]. The meta-analysis also involves emulsified fat, also called nanofat, containing m-SVF. An animal study conducted on rats demonstrated the effectiveness of m-SVF injected into the wound surface to expedite wound healing [41]. Additionally, a clinical trial illustrated the therapeutic effects of nanofat grafting on scars resulting from breast surgery [42]. Furthermore, a controlled trial involving both patients and rats compared standard fat grafting with standard fat grafting enriched with m-SVF, revealing not only the therapeutic benefits of standard fat grafting but also the superior scar reduction achieved with emulsified fat mixed fat grafting compared to simple fat grafting [43].

**Figure 7:**
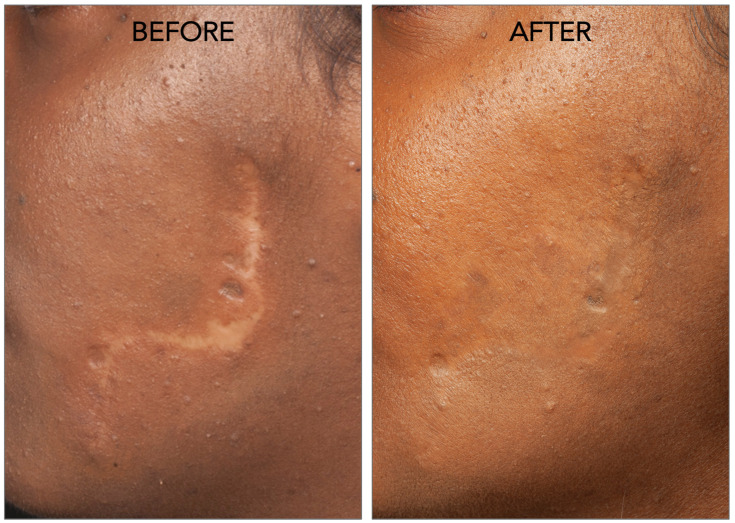
Fat grafting for reversing scars. The image represents an example of a facial scar treated with fat grafting by our team.

Regarding combined therapies, a trial indicated successful scar reduction with fat grafting combined with platelet-rich plasma (PRP) [44]. Similarly, a retrospective study affirmed the efficacy of nanofat grafting combined with fractional CO_2_ lasers in reducing depressed facial scars [45]. A representative image of scar reversal with fat grafting is illustrated in Figure 7.

### 4.2. Burns

Burns can lead to excessive scars, including keloids and hypertrophic scars, which result from aberrations in the process of physiologic wound healing. Post-burn scars stand as the predominant complication ensuing from a burn injury, with their severity contingent upon the depth of the burn. With the exception of superficial dermal burns, deeper burns typically heal through scarring. Despite efforts involving diverse physical therapy modalities and plastic surgical interventions, complete elimination of this scarring remains unattainable. This limitation impairs patients’ functional capabilities and adversely impacts their body image [46,47].

A systematic review of fat grafting for burn scars reports that several studies describe the treatment of burn scars with fat grafting [48]. One study reported a series of patients treated with two treatments of standard macro-fat grafting. All patients presented improvements in facial movements, skin texture, softness, thickness, and elasticity. The clinical results were supported by histological analysis, showing new collagen deposition, local hypervascularity, and dermal hyperplasia [49]. Similarly, another group reported clinical improvement in a series of patients with reductions in scar retraction, thickness of scar, and improvement in elasticity, and the clinical results were further confirmed by histological analysis showing neo-angiogenesis, collagen deposition, and dermal hyperplasia [50]. Other clinical studies showed a positive effect of fat grafting on the face and hands [51,52]. Studies conducted on mice experimentally demonstrated that conventional fat grafting expedites the process of revascularization at the burn site, as assessed through laser Doppler flow, *CD31* staining, and biomarkers of angiogenesis (*VEGF*), while concurrently reducing fibrosis, as indicated by Sirius red staining and biomarkers (*TGF-β* and *MMP9*) [53].

With regards to emulsified fat, multiple clinical studies have shown efficacy in improving skin stiffness and facial burn scars [54,55]. Another experimental study with a mouse model of third-degree burn showed that nanofat (m-SVF) was capable of enhancing wound closure, increasing neo-angiogenesis, accelerating the formation of granulation tissue, and boosting the thickness of the new epithelial layer [56].

Regarding SVF and ASCs, multiple clinical studies used e-SVF obtained with commercially available kits to treat burn scars, with positive results [57,58,59,60]. These clinical results were further confirmed by an experimental study on mice comparing fat grafting with ASCs in burn scars. The findings indicated a reduction in both burn wound depth and area in mice treated with fat grafting and/or ASCs. Moreover, the presence of apoptotic markers was notably diminished in mice receiving treatments incorporating ASCs [61].

### 4.3. Radiation-Induced Fibrosis (RF)

Radiation-induced fibrosis is caused by ionizing radiation used in the radiotherapy treatment of multiple types of cancer. It is due to a coordinated pathological process of wound healing, wherein radiation-induced endothelial dysfunction, persistent leukocyte infiltration, and abnormal extracellular matrix (ECM) deposition play pivotal roles. It has been considered a form of injury response where there is a continuous signal for connective tissue deposition and/or failure of the downregulatory processes that normally serve to terminate fibrogenesis; this perpetuation of stimuli elucidates why radiation fibrosis is a self-sustaining process that can endure for decades following radiotherapy and tends to exacerbate over time [62].

Rigotti first reported the successful treatment of irradiated tissues with fat grafting. Clinical improvements were confirmed by the tissue’s ultrastructural analysis, which revealed a reduction in collagen fibers in the connective tissue composing the irradiated tissues [63]. Multiple other clinical studies describe the successful application of lipotransfer in breast and head and neck irradiated fibrotic tissues [64,65,66,67,68,69,70,71]. An experimental study conducted on mice demonstrated that fat grafting mitigates inflammation in acute radiodermatitis and decelerates the advancement of fibrosis in chronic radiodermatitis. Specifically, fat injection resulted in reduced epidermal thickness and scar index [72]. Standard fat grafting was also compared with fat grafting enriched with ASCs to treat irradiated skin in mice, and results showed improved elasticity in irradiated wounds injected with fat + ASCs. Results showed that while fat grafts alone attenuated some of the pathologic changes associated with RT, fat grafting mixed with ASCs was found to significantly reduce skin stiffness, dermal thickness, and collagen content, returning measured levels of non-irradiated skin controls [73].

In vitro experiments revealed that ASCs significantly mitigated radiation-induced apoptosis by suppressing *CTSF* expression, subsequently downregulating pro-apoptotic proteins while upregulating anti-apoptotic ones. This suggests that ASCs offer protection against radiation-induced dermatitis by exerting an anti-apoptotic effect via *CTSF* inhibition [74]. In an animal study, the administration of SVF in rats demonstrated attenuation of radiation-induced skin injury, leading to improvements in wound healing and pain relief. Patients treated with SVF exhibited favorable skin texture and shape, with no recurrence of wounds [75]. Another study conducted on minipigs sought to compare the impact of adipose-derived stem cells (ASCs) and platelet-rich fibrin (PRF), either alone or in combination, on irradiated tissues. The findings revealed that both ASCs and PRF contribute to the healing of defects in irradiated minipigs, with their combined application proving more efficacious than when used individually [76].

### 4.4. Dupuytren’s Disease

Dupuytren’s disease (DD) is a fibroproliferative disorder characterized by abnormal deposition of fibrotic tissue in the palmar fascia of the hand. It arises either due to an impairment in the wound healing process or an abnormal response to injury. This condition is characterized by its chronic and progressive nature, which tends to deteriorate over time, leading to digital contracture. The pathogenesis of DD involves cellular events such as altered gene and protein expression of cytokines, growth factors, adhesion molecules, and components of the extracellular matrix, resulting in increased deposition of collagen III relative to collagen I levels [77,78]. The standard treatment involves surgical procedures like fasciotomy, fasciectomy, or dermofasciectomy [79]. Non-operative approaches, including the administration of clostridial collagenase injections, have also been explored, but their long-term efficacy remains limited [77,80,81].

Fat grafting has been successfully used in association with extensive percutaneous aponeurotomy for the treatment of Dupuytren’s contracture. Khouri’s group treated 99 hands after performing the percutaneous aponeurotomy. The outcome showed improvement in contracture rate in interphalangeal and metacarpophalangeal joints [82]. The same group demonstrated in an experimental study that the ASCs are able to inhibit the contractile DD’s myofibroblast. They conducted a study where Dupuytren’s myofibroblasts were co-cultured with either adipose-derived or bone marrow-derived stem cells to evaluate isometric force contraction. They measured the levels of α-smooth muscle actin mRNA and protein expression. The proliferation of Dupuytren’s myofibroblasts was also assessed. The results indicated that the addition of adipose-derived stem cells to Dupuytren’s myofibroblasts decreased their contraction, leading to a reduction in α-smooth muscle actin protein expression, and inhibited their proliferation [83].

### 4.5. Lichen Sclerosus

Lichen sclerosus is a chronic inflammatory disorder affecting the genital and perianal areas. It is a relatively common condition affecting circa 3% of women, and it is caused by a chronic autoimmune response, leading to dermal hyperkeratosis, skin fibrosis, and reduced tissue elasticity. Symptoms of the condition include itching, a burning sensation, soreness, and pain. The presence of erosions, fissures, or introital narrowing can result in significant and debilitating dyspareunia, leading to sexual dysfunction [84]. In more advanced stages, fissures and tears may develop, and the resulting scarring can cause anatomical changes that, if left untreated, may become irreversible and lead to the loss of vulvar architecture. Despite its widespread use in reconstructive surgery, the application of autologous fat grafting in LS is relatively new. The objective is to ameliorate the fibro-sclerotic manifestations of LS, thereby improving symptoms, sexual function, and overall quality of life for patients.

Multiple studies implemented standard fat grafting to treat LS [85,86,87,88,89,90,91,92]. The bioproduct used for injection was standard fat grafting [85,86], fat grafting mixed with PRP [87] or emulsified fat [88,90], emulsified fat mixed with PRP [89,92], and laboratory-expanded ASCs vehiculated in a scaffold of hyaluronic acid [91]. In all studies, symptoms and overall quality of life, including sexual function, were reported to be ameliorated.

Clinical results were corroborated by histologic analysis in two studies. In one of the studies, standard fat grafting was used, and it was detected that there was a marked reduction in hyperkeratosis in 67% of patients, a reduction in chronic inflammation in 89% of the cases, and a reduction in fibrosis in 67% [86]. The second study implemented ASCs in HA scaffold and reported that dermis sclerosis was significantly reduced, capillaries were less dilated, and inflammatory infiltrate was dramatically reduced [91].

### 4.6. Scleroderma

Scleroderma is a chronic fibrotic autoimmune disorder due to autoantibodies against the microvasculature affecting the connective tissue of the skin and internal organs. It is characterized by thickening and fibrosis due to abnormal deposition of ECM, in particular type I collagen [93]. The affected patients may present loss of elasticity and tightness of the skin in a localized area, or it can be systemic, manifesting around the lips with microstomia, eating and speaking limitations, or in the fingers with sclerodactyly and vascular complications leading to functional impairment [94]. Fat grafting has been used both in localized scleroderma to correct deformity and replace volumes and in systemic sclerosis to ameliorate skin fibrosis.

Standard fat grafting was adopted in multiple studies involving scleroderma patients [95,96,97,98,99,100,101,102]. In one study, standard fat grafting was enriched with previously harvested and laboratory-expanded ASCs [101]. Another study mixed ASCs with PRP [102]. All studies reported improvement not only in mouth opening but also in patients’ quality of life. Punch biopsies were also performed, showing improvement in skin keratosis and fibrosis, dermo-epidermic junction flattening, and microvascular density [98,99].

In an experimental study on scleroderma-induced mice, the effects of BMSCs and the ASCs injected intravenously were compared. Skin thickness, histology, immunostaining, collagen determination, and RT-qPCR were performed. Compared to the BMSCs, the ASC were significantly more efficient in reducing skin fibrosis, which was related to a stronger reduction in *TNFα*, *IL1β*, and an enhanced ratio of *MMP1/TIMP1* in both skin and lung tissues [103]. In another animal study, standard fat grafting was compared to micro-fat grafting, SVF, and PRP. The researchers found that combinations such as micro-fat grafting with SVF and micro-fat grafting with PRP effectively reversed both dermal and epidermal sclerosis. However, they observed that standard fat grafting alone, SVF, and PRP were only able to correct dermal sclerosis [104].

The effect of the different adipose stem cells-based therapies successfully used to reverse dermal fibrosis and scarring in multiple fibrotic skin conditions are summarized in Table 1.

## 5. Discussion

### 5.1. Mechanism of Action of ASCs-Based Therapies in Dermal Fibrosis

**Figure 8:**
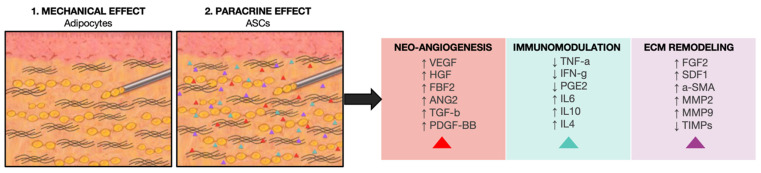
Mechanism of action. The diagram illustrates how fat grafting and ASCs-based therapies reduce scarring and skin fibrosis with a combination of mechanical (1) and paracrine (2) effects. The latter is mediated mainly by the ASCs, which can release cytokines and growth factors with pro-angiogenetic, immunomodulatory, and trophic effects.

There is certainly a direct mechanical effect by injecting adipose tissue into fibrotic skin, as the adipocytes exert a padding effect and dilute the tethering collagen fibers within the fibrotic or scarred tissue, reducing its stiffness (Figure 8 (1)). However, the main anti-fibrotic therapeutic effect is due to a regenerative effect, which is mainly driven by the ASCs, either extracted and expanded in culture or as they are in the lipoaspirate or SVF (Figure 8 (2)). Experimental studies have delved into the mechanisms underlying the reduction of fibrosis by ASCs, revealing a combination of various modes of action, including stimulation of neo-angiogenesis, modulation of the immune response, and trophic effects on the ECM (Figure 8).

Fibrosis is defined by an excessive buildup of extracellular matrix (ECM) and its insufficient breakdown. The increased ECM synthesis is primarily orchestrated by *TGF-β1*, while the degradation of ECM is governed by an equilibrium between *MMPs* and *TIMPs*. ASCs have the ability to suppress ECM generation and facilitate the breakdown of its constituents. The main anti-fibrotic effect of ASCs is exerted through the inhibition of *TGF-β1*, one of the principal pro-fibrotic molecules involved in skin fibrosis. 

*TGF-β1* is primarily synthesized by T-cells during the healing process and becomes activated through the actions of *MMPs*, reactive oxygen, and nitrogen species (ROS and RNS), as well as various cytokines. Furthermore, *TGF-β1* has been observed to downregulate the expression and activity of *MMPs* while promoting the expression of *TIMPs* [105]. ASCs have been suggested to diminish the expression of *TGFβ1* and its downstream target genes, including collagen type I, type III, and α-smooth muscle actin (*α-SMA*), by secreting *HGF*. Multiple studies have demonstrated the in vitro and in vivo ability of ASCs to downregulate *TGF-β1*. In a co-culture experiment, our team observed that the secretion of *TGF-β1* was notably reduced in the presence of adipose-derived stem cells (ASCs) when fibroblasts from systemic sclerosis patients were co-cultured with ASCs compared to fibroblasts cultured alone. This suggests that ASCs may exert an inhibitory effect on fibrosis through paracrine signaling mechanisms. [95]. Other researchers have shown that adipose-derived stem cells (ASCs) alleviate radiation-induced muscular fibrosis by suppressing the expression of *TGF-β1* in a rabbit model [106]. Additionally, ASCs have been demonstrated to reverse vocal fold scarring by suppressing *TGF-β1* signaling in vitro [107]. The reduction in activated *TGF-β1* levels results in decreased proliferation of myofibroblasts, thereby altering the equilibrium between ECM synthesis and degradation. However, the precise mechanism underlying this effect remains unclear [105]. Another suggested anti-fibrotic mechanism involves the suppression of connective tissue growth factor (*CTGF*), which collaborates with *TGF-β1* to enhance fibroblast proliferation, migration, and adhesion, as well as extracellular matrix production [95]. Another important role of the ASCs is their effect on the ECM. In the process of wound healing, the provisional extracellular matrix (ECM) undergoes degradation facilitated *MMPs* once the tissue replacement is complete. The delicate balance between MMPs and their inhibitors, known as *TIMPs*, regulates the accumulation of ECM, and any disruption in this *MMP/TIMP* ratio can lead to fibrosis. Evidence showed that ASCs tend to upregulate *MMP-1, -3, MMP-2* and the *MMP-2/TIMP-2* ratio, remodeling the fibrotic extracellular matrix [105].

Another interesting feature of ASC therapy is its immunomodulatory ability. By reducing the production of proinflammatory cytokines such as *TNF-α* and *IFN-γ*, adipose-derived stem cells (ASCs) establish a “virtuous circle” wherein fewer immune cells migrate to damaged tissues. This inhibition of the acute inflammatory reaction and cytokine production by ASCs contributes to a decrease in subsequent chronic inflammation and fibrosis. Moreover, the alleviation of tissue inflammation, enhancement of angiogenesis, and mitigation of oxidative stress further enhance their anti-fibrotic efficacy [108,109].

### 5.2. Surgical Variability

Different adipose stem cell-based therapies are available, and each is associated with advantages and disadvantages. The use of autologous standard fat grafting presents multiple advantages, including sound evidence on its safety and efficacy in terms of anti-fibrotic effect; adipose tissue is easily available; the procedure is minimally invasive; it is effective both for volumetric effect and tissue regeneration; and the overall procedure is in compliance with current regulation in multiple countries. The disadvantages of using fat grafting consist of the need to repeat the procedure, potential resorption and partial volume loss over time, unclear durability of the results, general anesthesia/sedation required, risk of lumpiness and asymmetry, and a downtime of 1–2 weeks. Various authors have employed different surgical techniques for the aspiration, processing, and injection of adipose tissue, potentially influencing the cellular composition selected during fat preparation. One of the most commonly used surgical techniques is the method described by Sydney Coleman [36]. This method entails gently and atraumatically aspirating subcutaneous adipose tissue using a 3 mm-diameter suction cannula connected to a 10 cm^3^ syringe. After centrifugation at 1200× *g* for 3 min, the processed adipose tissue is injected subcutaneously using a 17 G cannula connected to a 1 cm^3^ syringe.

Certain studies applied the Coleman techniques with minor modifications (infiltration, fat harvesting, time of centrifugation, and size of cannulas/syringes). Other authors preferred not to centrifuge the fat, and they processed the adipose tissue either by decantation or filtration. Recently, the micro-fat grafting technique has been introduced to obtain smaller fat lobules compared to the ones obtained with the Coleman technique (around 1 mm), allowing a more superficial injection [104,110]. The approach entails a gentle liposuction using a 1 mm-diameter micro-blunt cannula with multiple holes attached to a 10 cm^3^ syringe, followed by a brief centrifugation at 1200× *g*/min for 2 min. The resulting small fat lobules, approximately 500 μm in size, can then be reinjected using a 21 G cannula (0.8 mm) [104,110].

Despite these procedural differences, all studies reported notable improvements post-treatment. Using ASCs offers several advantages, including their potential to differentiate into various cell types, abundant secretion of growth factors such as *VEGF*, *HGF, FGF-2*, and *IGF-1*, immunomodulatory properties, and induction of tolerance [111].

Instead, the use of ASCs requires a liposuction procedure prior to the primary injection, and ASC expansion also requires cultivation procedures at cell facilities that are currently costly, time-consuming, and not in compliance with regulatory bodies in multiple countries. The advantages of using SVF lie in its easy accessibility and extraction directly in the operating room, streamlining the treatment to a single procedure [112]. Consequently, surgeons frequently opt for the application of mechanically extracted SVF, also known as nanofat or emulsified fat, over adipose-derived stem cells (ASCs) [37]. While ASCs constitute a homogeneous cell population devoid of cells like leukocytes and endothelial cells, SVF cell preparations represent a heterogeneous mixture consisting of cell debris, perivascular cells, inflammatory cells (such as leukocytes), endothelial cells, and erythrocytes. Consequently, the latter exhibits increased immunogenicity compared to ASCs [113]. However, the application of ASCs- or SVF-based cell therapy in clinical practice is hindered by the relatively low number of published clinical studies and the absence of standardized protocols.

### 5.3. Limitations of the Application of ASCs

Cell-based therapies are a cutting-edge method for treating diseases for which there are few or no viable treatment choices. Nevertheless, there are limitations to the application of ASCs. First, the durability of the effect is unclear. Part of the grafted fat and ASCs do not survive after the injection, but their secretome creates a microenvironment, contributing to intrinsic recovery. In some chronic conditions, like scleroderma, it is difficult to assess the durability of the effect as the disease is progressive; therefore, the treatment often needs to be repeated. Other limitations on the application of ASCs include the variability among different patients regarding age, body mass index (BMI), and health status (underlying disease or comorbidities), which may lead to diminished capacities of ASCs’ key regulatory factors. Variations in fat injection technique, including factors such as syringe size, cannula size, injection rate, volume of injection, and others, may have a significant impact on volume retention and the regenerative outcome.

## 6. Conclusions

There is substantial evidence that adipose stem cell-based therapy for fibrosis and scarring is effective in modifying scar tissue and fibrosis. Both human and animal studies demonstrated that fat grafting and cell therapy with ASCs present interesting potentialities for the treatment of different fibrotic conditions. Through softening fibrosis, function and overall quality of life may be considerably enhanced in different patient cohorts.

The anti-fibrotic effect is likely primarily mediated by ASCs, which are abundant and easily expandable cells capable of undergoing various types of differentiation in vitro, including adipogenic, osteogenic, chondrogenic, neurogenic, and myogenic differentiations. However, the exact mechanism responsible for their anti-fibrotic effect remains unclear. Certainly, it encompasses an intricate interplay among various cellular constituents, involving adipocytes, adipose-derived stem cells (ASCs), pericytes, and biological molecules like cytokines and growth factors. These elements are accountable for fostering angiogenesis, modulating the immune response, and exerting trophic effects. To date, it is unclear whether standard fat grafting is more effective alone or in combination with PRP or ASCs. In summary, while these emerging therapies show promise, there is a requirement for randomized controlled trials and quantitative analysis to substantiate their efficacy and cost-effectiveness in managing fibrosis and scarring.

## Figures and Tables

**Table 1:** The table summarizes the effects of the adipose-derived stem cell-based therapies (ASCs-BT) on each condition.

Condition	Etiology	Type of ASCs-BT	Proposed Effect	References
Hypertrophic scar	Trauma; Iatrogenic	Macro-fat; Emulsified fat/m-SVF; Macro-fat mixed with PRP; Emulsified fat/m-SVF combined with CO_2_ laser	ECM remodeling	[40,41,42,43,44,45]
Burns	Trauma	Macro-fat; Emulsified fat/m-SVF; e-SVF	ECM remodeling; Neo-angiogenesis	[48,49,50,51,52,53,54,55,56,57,58,59,60,61]
Radiation-induced fibrosis	Iatrogenic	Macro-fat	ECM remodeling; Neo-angiogenesis; Immunomodulation; Mechanical effect: increase in thickness of subcutaneous tissue	[63,64,65,66,67,68,69,70,71,72,73,74,75,76]
Dupuytren’s disease	Unknown (?genetic)	Macro-fat; ASCs	Myofibroblast proliferation inhibition; α-smooth muscle actin protein expression reduction; Angiogenesis; Immunomodulation	[82,83]
Lichen sclerosus	Unknown (?genetic)	Macro-fat; Emulsified fat/m-SVF; Fat mixed with PRP; ASCs	ECM remodeling; Immunomodulation	[85,86,87,88,89,90,91,92]
Scleroderma	Autoimmune	Macro-fat; Micro-fat; Emulsified fat/m-SVF; ASCs; ASCs mixed with PRP; Micro-fat mixed with m-SVF; Micro-fat mixed with PRP; Emulsified fat/m-SVF mixed with PRP	ECM remodeling; Neo-angiogenesis; Immunomodulation	[95,96,97,98,99,100,101,102,103,104]
